# Tooth Wear and Salivary Factors: Insights from a Cohort of Dental Students

**DOI:** 10.3390/jcm14061936

**Published:** 2025-03-13

**Authors:** Manuel Nobre, Laura Almeida, Madalena Magalhães, Rui Carvalho, João Rua, Luís Proença, Ana M. Vieira

**Affiliations:** 1 Clinical Research Unit (CRU), Egas Moniz Center for Interdisciplinary Research (CiiEM), Egas Moniz School of Health & Science, 2829-511 Almada, Portugal; rpmcarvalho@egasmoniz.edu.pt (R.C.); jrua@egasmoniz.edu.pt (J.R.); lproenca@egasmoniz.edu.pt (L.P.); asvieira@egasmoniz.edu.pt (A.M.V.); 2 Egas Moniz University Clinic, Egas Moniz School of Health & Science, 2829-511 Almada, Portugal; laura.seidenader@gmail.com (L.A.); madalenadsmagalhaes@gmail.com (M.M.)

**Keywords:** erosion, tooth wear, BEWE index, salivary flow, salivary pH, risk factors

## Abstract

**Background/Objectives**: Tooth wear is a progressive and multifactorial condition influenced by mechanical and chemical factors. Saliva plays a crucial role in modulating erosive wear through its buffering capacity and remineralization potential. This study aimed to assess the prevalence and severity of erosive tooth wear among dental students and explore its correlation with salivary factors such as flow rate and pH. **Methods**: A cross-sectional study was conducted on 96 individuals from the Egas Moniz School of Health & Science. Erosive wear was evaluated using the Basic Erosive Wear Examination (BEWE) index. Unstimulated and stimulated saliva samples were collected to measure salivary flow rate and pH. Statistical analyses included Spearman’s correlation and multinomial logistic regression. **Results**: All individuals were classified as no risk (BEWE Score 0–2) or low risk (BEWE Score 3–8). The most affected teeth were the lower first molars and the 4th and 6th sextants. Salivary analysis showed a mean unstimulated flow rate of 0.5 mL/min and stimulated flow of 1.7 mL/min. A significant negative correlation (rho = −0.224, *p* = 0.029) was found between stimulated salivary pH and BEWE score, indicating that higher acidity contributes to greater erosive wear. **Conclusions**: This study highlights the impact of salivary properties on erosive tooth wear, emphasizing the protective role of higher salivary pH. Regular monitoring of saliva and preventive strategies should be integrated into early diagnosis and management of erosive wear in young adults.

## 1. Introduction

Tooth wear is defined as the progressive loss of dental hard tissue through mechanical, chemical, or combined mechanisms, independent of dental caries. Tooth wear can occur through various mechanisms, including attrition, abrasion, and erosion [1,2,3,4]. This phenomenon is universally recognized as a physiological process observed across all civilizations and historical periods. Once teeth erupt and establish contact with opposing dentition, they become subject to wear. Studies suggest that this process is continuous, characterized by phases of varying intensity over time [5,6].

Erosive tooth wear is defined as the irreversible loss of hard dental tissues caused by chemical demineralization due to non-bacterial acids. This phenomenon can affect both primary and permanent dentition and is associated with intrinsic acids, such as gastric juice in cases of gastroesophageal reflux, vomiting, or eating disorders, as well as extrinsic acids from acidic diets, medications, and behavioral habits. Erosion leads to the loss of dental morphology, characterized by smooth, polished surfaces, rounded cusps, and occlusal cupping, which compromise dental function and esthetics [7,8].

The progression of erosive wear depends on the frequency, duration, and intensity of acid exposure, as well as individual susceptibility factors, including salivary flow rate, buffering capacity, and salivary composition. Saliva plays a critical role in neutralizing acids and remineralizing dental surfaces, although its protective effect may be limited under frequent and intense acidic challenges. Additionally, the acquired pellicle provides a protective barrier that reduces demineralization and facilitates remineralization [9,10].

The prevalence of erosion in the 18–25 age group in Europe is 26.5%. When considering prevalence in Spain (a Mediterranean country) for the 18–35 age group, the rate is 26.3% [11].

Early diagnosis of erosive tooth wear is essential and involves identifying morphological changes and assessing patients’ dietary and behavioral habits. The Basic Erosive Wear Examination (BEWE) is the most widely used and recommended index for this purpose. BEWE evaluates the severity of erosive wear in each dental sextant using a scale from 0 (no wear) to 3 (loss of more than 50% of the surface). The sum of the scores across all sextants generates a total BEWE score, which guides the assessment of erosive risk and the implementation of preventive and therapeutic measures. These measures range from routine clinical monitoring and fluoride application to restorative interventions in severe cases [12].

This study aims to evaluate the prevalence and distribution of erosive tooth wear in a sample of students from the Egas Moniz Dental Clinic using the BEWE index, correlating the BEWE score with the flow rate of stimulated and unstimulated saliva, as well as assessing the role of salivary pH in erosive tooth wear.

The relevance of this research lies in its study population, as dental students represent a homogeneous group with controlled variables, minimizing confounding factors such as inconsistent oral hygiene habits or varying access to dental care. This controlled setting facilitates a more precise analysis of the specific impact of salivary factors, such as pH and flow rate, on erosive wear, offering a clearer understanding of their role in erosion progression.

By focusing on this population, the study provides valuable insights into the early manifestations of erosive wear, contributing to a deeper understanding of its risk factors and supporting the development of targeted preventive strategies for at-risk individuals.

Such measures are critical to controlling the progression of erosive wear and preserving oral health [9,12].

## 2. Materials and Methods

### 2.1. Ethical Approval

This study was approved by the Ethics Committee of the Egas Moniz School of Health and Science (Process No. 717). All participants were informed of the research purpose both verbally and in writing. They provided written informed consent, ensuring anonymity and confidentiality of the data and their right to withdraw at any time. The study was conducted at the Egas Moniz Dental Clinic over a two-month period, between June and July 2019.

### 2.2. Patient Selection

Participants included 102 participants (students and alumni) of the Integrated Master’s in Dental Medicine program at Egas Moniz School of Health and Science, aged between 19 and 36 years. The sample was randomly selected, and 6 participants were excluded for having fixed orthodontic appliances such as brackets, bands, or attachments, resulting in a final sample of 96 individuals (*n* = 96). Fixed retention devices were not considered exclusion criteria. Each participant was assigned a unique identification code to ensure anonymity, and data were recorded centrally and securely.

### 2.3. Study Protocol

Prior to the clinical study, the investigator was trained and calibrated on the BEWE index in collaboration with an experienced investigator (A.V.). Calibration was performed by observing seven individuals independently, with both observers achieving substantial inter-rater agreement (Cohen’s kappa = 0.649, *p* < 0.05) following Landis & Koch (1977) [13] criteria. After calibration, the investigator independently examined and collected data from the study participants. 

Saliva collection followed standardized protocols described in the literature [14,15]. Participants were instructed to abstain from eating, drinking, smoking, or practicing oral hygiene for 60 min prior to saliva collection.

Unstimulated saliva was collected with participants seated upright in a relaxed position. Participants were instructed to swallow saliva present in their oral cavity and then let saliva drip naturally into a collection cup without spitting or stimulating salivary flow by any means. The collection period was 3 min.

Stimulated saliva was collected after participants chewed paraffin gum (CRT Refill, Ivoclar Vivadent Clinical, Schaan, Lichtenstein) for one minute. They were then asked to let saliva drip into the collection cup during a 3 min period. The volume of saliva collected for both unstimulated and stimulated conditions was measured using a 5 mL graduated syringe (Single Use Syringe, Henry Schein, Orange, CA, USA). Salivary flow rate was calculated by dividing the volume of saliva by the collection time.

Immediately after saliva collection, the pH of both unstimulated and stimulated saliva was measured using colorimetric pH test strips (HYDRION^®^, Micro Essential Laboratory Inc., Brooklyn, NY, USA), following the manufacturer’s instructions.

The clinical examination assessed erosive dental wear using the BEWE index [12]. Deciduous teeth, crowns, and implants were excluded from the evaluation. The examination was performed using sterile basic diagnostic kits, including tweezers, a mirror, and an exploratory probe. Participants were seated in a dental chair equipped with an overhead light for optimal visualization. A three-way syringe was used to lightly dry tooth surfaces before assessment.

The BEWE index scores dental wear on a scale of 0 to 3:

- 0: No erosive tooth wear;
- 1: Initial loss of surface texture;
- 2: Distinct defect, hard tissue loss < 50% of the surface area;
- 3: Hard tissue loss ≥ 50% of the surface area.

The highest score from each sextant was recorded, and the total BEWE score was calculated by summing the sextant scores. Risk levels were determined as shown in Table 1.

### 2.4. Statistical Analysis

The BEWE index was defined as the primary dependent variable. The following independent variables were considered: age, gender, salivary flow rate, and pH (stimulated and unstimulated).

The crude relationship between the continuous independent variables and the BEWE index was examined by correlation analysis (Spearman’s rank correlation). Afterwards, stepwise multinomial logistic regression procedures were used to evaluate and model the impact of the considered independent variable on the BEWE index.

Statistical analyses were performed by using IBM SPSS Statistics v.29 (Armonk, NY, USA). A 5% significance level was established in all inferential analyses.

## 3. Results

### 3.1. Sample Characterization

A total of 96 young adults, aged between 19 and 36 years, with a mean age of 24.3 (±3.1) years, were observed. It was found that 71 participants (74.0%) were female, and 25 (26.0%) were male.

### 3.2. BEWE and Erosive Tooth Wear

The BEWE index/score ranged from 0 to 8, with a mean value of 1.6 (±1.8). A score of 0 was the most prevalent, being observed in 34 individuals (35.4%), while higher scores were much less frequent, with only one participant scoring 7 and another scoring 8.

Analyzing erosive tooth wear in BEWE score groups, it was found that 73 individuals (76.0%) had a BEWE score below 2, classifying them as having no risk. The remaining 23 individuals (24.0%) had a BEWE score of 3 or higher but less than 9, belonging to the low-risk group (Table 2).

Regarding the degrees of tooth wear observed, only 15 individuals (15.6%) showed a grade 2 wear in their teeth, while the remaining 81 individuals (84.4%) had lower grades (0 and 1).

When analyzing the degree of erosive tooth wear in individual teeth, it was observed that 2407 teeth (90.0% of the teeth examined) had a grade 0. Grade 1, characterized by initial enamel structure loss, was present in 237 teeth (9.0%). Grade 2, observed in 23 teeth (1.0%), was associated with distinct defects and loss of less than 50% of hard dental tissues, enamel, and dentin.

This highest grade was primarily observed in lower first molars, with nine first molars in the 4th sextant and 10 first molars in the 6th sextant (Table 3).

### 3.3. Salivary Parameters

The salivary flow rate of each participant was recorded and classified as “very low” (<0.1 mL/min), “low” (0.1–0.25 mL/min), and “normal” (>0.25 mL/min) for unstimulated saliva and as “very low” (<0.7 mL/min), “low” (0.7–1.0 mL/min), and “normal” (>1.0 mL/min) for stimulated saliva.

Among the 96 individuals observed, one was unable to produce unstimulated saliva, resulting in a sample size of *n* = 95 for salivary flow and pH analysis.

The mean value of unstimulated salivary flow was 0.5 (±0.4) ml/min, while for stimulated salivary flow, the mean value was 1.7 (±1.0) ml/min. Most individuals exhibited a normal salivary flow (74% for unstimulated saliva and 71% for stimulated saliva).

The pH of unstimulated saliva ranged from 4.0 to 9.0, with a mean value of 7.0 (±0.91). The stimulated saliva pH had a mean value of 8.19 (±0.79), with a range of 6 to 10 (Table 4 and Table 5).

Within the group of patients with very low/low salivary flow, the prevalence of dental erosion is 72% for unstimulated saliva and 71.42% for stimulated saliva.

### 3.4. Relationship Between Sociodemographic and Clinical Variables and BEWE Index

The relationship between age and salivary parameters and the BEWE index was evaluated by Spearman’s correlation analysis. A significant negative weak correlation was identified between the stimulated salivary pH and BEWE index (rho = −0.224, *p* = 0.029). Additionally, the BEWE index was not found to significantly differ between males and females (*p* = 0.372, Mann–Whitney test) as in Table 6.

### 3.5. Modeling the Impact of the Studied Variables on BEWE Index

By using a stepwise multinomial regression procedure, it was possible to evaluate the overall joint impact of the considered independent variable on the BEWE index. The final reduced model identified stimulated salivary pH as the sole significant predictor towards the BEWE index (B = −0.511, *p* = 0.036).

## 4. Discussion

This study aimed to evaluate the prevalence of erosive tooth wear among students and alumni at the Egas Moniz Dental Clinic using the BEWE index and to explore its relationship with salivary risk factors, such as pH and salivary flow rate. Erosive tooth wear has gained increasing importance in clinical practice and research, driven by lifestyle changes and greater exposure to causative agents. Its multifactorial nature makes accurate diagnosis a cornerstone for effective prevention and treatment strategies [16].

The BEWE index, introduced in 2008, has emerged as a practical and reliable tool for assessing erosive tooth wear. Endorsed by the European Federation of Conservative Dentistry, it meets the scientific community’s need for a standardized and efficient method to quantify risk. Unlike earlier indices, BEWE facilitates cross-study comparisons and integrates seamlessly with existing data, further solidifying its value in both clinical and research settings [4,12,17,18]. The high sensitivity and specificity of the BEWE index, as demonstrated in multiple studies, supports its use in prevalence studies and in the classification of erosive lesions [11,19]. The calibration of examiners, as performed in this study, ensures consistent and reproducible results, with the substantial agreement observed (k = 0.649; *p* = 0.0001) aligning well with prior research [11,16,17,19,20].

The findings revealed that most participants (76%) had no risk of erosive wear (BEWE score ≤ 2), while 24% were categorized as low risk (BEWE score 3–8). Moderate to severe wear (grades ≥ 2) was observed in 15.6% of the sample. These results are consistent with studies in Mediterranean populations, such as Spain (26.3%) and Italy (21.9%), suggesting a similar pattern of wear influenced by cultural and dietary habits [11].

In a different study conducted at the same university clinic, but in a population of high-performance athletes, 64% of patients were classified as no-risk, a percentage similar to that found in the present study. However, unlike our findings, moderate- and high-risk cases were also observed in the athlete population.

It is important to highlight that this study considered additional factors that were not assessed in the present research, such as dietary habits and oral hygiene practices. In high-performance sports, the frequent consumption of low-pH beverages can significantly influence the rate of dental erosion, which may explain the higher presence of moderate- and high-risk cases in that population [21].

The lower first molars, particularly in sextants 4 and 6, were identified as the most affected teeth, a pattern consistent with previous research. Some studies have also reported significant wear on the palatal surfaces of maxillary teeth, which did not occur in the present study [11,15,19,22]. Among other factors, this may be due to the type of saliva present in the region. Both the occlusal surfaces of the lower molars and the palatal surfaces of the upper incisors are covered by a layer of mucous saliva, which has a lower buffering capacity [23].

The BEWE index simplifies the classification of dentin exposure, focusing on the lesion’s extent across the tooth surface rather than its depth. This approach addresses the diagnostic challenges highlighted by Ganss et al. (2006) and Mulic et al. (2010), where visual assessments often struggle to determine the severity of lesions involving dentin [19,24]. By emphasizing surface involvement, the BEWE index ensures a more comprehensive evaluation of erosive lesions.

Unlike what might be expected, no significant relationship/impact was found between gender, age, and the prevalence of erosive wear, consistent with Vered et al. (2014) [16]. This reinforces the notion that individual factors such as salivary properties and dietary habits may outweigh demographic influences. However, the smaller sample size in this study, compared to Vered et al., suggests that larger-scale investigations could provide additional insights [16].

Saliva plays a vital protective role in mitigating erosive risk. Its properties, such as pH buffering capacity and flow rate, are critical in maintaining oral health. Salivary diagnostics offer a quick and non-invasive method for evaluating these parameters, making them highly valuable for identifying at-risk individuals and monitoring their condition. [25,26]. Although not yet a routine tool in clinical practice, sialometry is particularly beneficial for diagnosing conditions like xerostomia and hyposalivation [4].

A recent investigation by Madariaga (2025) explored the relationship between salivary pH, salivary flow rate, and the severity of tooth wear, utilizing a distinct methodological approach and employing indices different from those used in the present study [27]. Despite these methodological differences, Madariaga’s findings align closely with those of the current research, highlighting an association between stimulated salivary pH and the severity of tooth wear. This underscores the importance of salivary pH as a critical protective factor against acid-induced tooth wear. Furthermore, it may indicate that the monitoring of saliva pH may be used as a risk assessment tool in erosive tooth wear [28].

Although the BEWE index is a validated and widely used method for the clinical assessment of erosive wear, more recent approaches, such as the use of intraoral scanners, enable longitudinal monitoring and the detection of minimal morphological changes. The combined use of both methods allows for a more precise and earlier preventive approach [29].

The average timeframe required to observe erosive changes using indices is approximately 18 months. Therefore, it is crucial to develop methods capable of reducing this diagnostic interval. According to O’Toole et al. (2023), while qualitative tools like study models and clinical photographs often require up to two years to detect changes, clinical indices such as the Basic Erosive Wear Examination (BEWE) can identify alterations within this 18-month window [30]. Furthermore, integrating digital 3D imaging with the BEWE index, as highlighted by Marro et al. (2018) [31], enhances early detection, with intraoral scanners showing potential to monitor changes in as little as six months. This increased sensitivity to initial changes enables earlier intervention and the implementation of preventive strategies, particularly in younger populations where the prevalence of erosive tooth wear continues to rise [31].

Nevertheless, this study faced several limitations. The primary constraints were the small sample size and the homogeneity of participants, predominantly young adults studying dentistry. This demographic likely contributed to a lower prevalence of risk factors and a heightened awareness of oral health behaviors. Additionally, the saliva collection method and pH testing procedures may have influenced sample quality. For instance, the exposure of saliva to the environment may have led to CO_2_ loss, potentially causing an artificial increase in pH values.

Despite these limitations, this study provides valuable insights into the interplay between erosive wear and salivary factors. It highlights the necessity for further research involving larger and more diverse populations to better understand these relationships and validate the findings in broader contexts.

## 5. Conclusions

According to the results obtained and acknowledging the limitations of this study, it was possible to conclude that 76% of participants presented no risk (BEWE Score 0–2) whilst 24% of the participants presented low risk of erosive wear (BEWE Score 3–8), being the most affected teeth the lower molars.

Regarding risk factors, saliva was observed to play a crucial protective role against dental erosion. Therefore, monitoring salivary parameters, such as salivary flow rate and pH, especially when stimulated, is essential for early detection and prevention strategies.

Despite the relevance of these findings, it is important to acknowledge that the study sample was small and highly homogeneous, consisting of individuals with high oral health literacy, which may limit its representativeness for a broader population. Future studies should include larger and more diverse samples, incorporating additional variables to enhance the understanding of risk factors associated with erosive tooth wear.

Dental professionals play a fundamental role in preventing and detecting early signs of erosive wear. Therefore, the integration of standardized indices, such as the BEWE index, into routine dental examinations is crucial for improving diagnostic accuracy and implementing effective preventive strategies.

## Figures and Tables

**Table 1:** BEWE score classification by risk level.

Risk	BEWE Score
No risk	0–2
Low risk	3–8
Medium risk	9–13
High risk	≥14

BEWE: Basic Erosive Wear Examination.

**Table 2:** Distribution of risk score among participants, with absolute numbers (*n*) and percentages (%).

Risk	*n*	%
No risk	73	76
Low risk	23	24
Medium Risk	0	0
High risk	0	0

**Table 3:** Distribution of BEWE score by sextant among participants, with absolute numbers (*n*) and percentages (%).

Erosive Tooth Wear Grade	1st Sextant		2nd Sextant		3rd Sextant		4th Sextant		5th Sextant		6th Sextant	
	*n*	%	*n*	%	*n*	%	*n*	%	*n*	%	*n*	%
0	84	88	70	73	76	79	56	58	89	93	67	70
1	10	10	26	27	19	20	31	32	7	7	18	19
2	2	2	0	0	1	1	9	10	0	0	11	11
3	0	0	0	0	0	0	0	0	0	0	0	0

**Table 4:** Distribution of unstimulated saliva flow rates among participants, with absolute numbers (*n*) and percentages (%).

Unstimulated Saliva Flow					
Very Low (<0.1 mL/min)		Low (0.1–0.25 mL/min)		Normal (>0.25 mL/min)	
*n*	%	*n*	%	*n*	%
5	5	20	21	71	74

**Table 5:** Distribution of stimulated saliva flow rates among participants, with absolute numbers (*n*) and percentages (%).

Stimulated Saliva Flow					
Very Low (<0.7 mL/min)		Low (0.7–1.0 mL/min)		Normal (> 1.0 mL/min)	
*n*	%	*n*	%	*n*	%
7	7	21	22	68	71

**Table 6:** Correlation coefficients (rho) and *p*-values (*p*) for the association between age, salivary parameters, and the studied outcome.

	rho	*p*
Age	−0.019	0.854
Salivary flow rate (unstimulated)	−0.098	0.342
Salivary flow rate (stimulated)	−0.103	0.317
Salivary pH (unstimulated)	−0.158	0.127
Salivary pH (stimulated)	−0.224	0.029

## Data Availability

The original contributions presented in the study are included in the article and further inquiries can be directed to the corresponding authors.

## Abbreviations

The following abbreviations are used in this manuscript:

BEWE	Basic Erosive Wear Examination
